# Wind and solar energy in Small Island Developing States for mitigating global climate change

**DOI:** 10.1016/j.isci.2024.111062

**Published:** 2024-09-27

**Authors:** Peni Hausia Havea, Buda Su, Changyi Liu, Zbigniew W. Kundzewicz, Yanjun Wang, Guojie Wang, Cheng Jing, Han Jiang, Fang Yang, Fiamē Naomi Mata’afa, Tong Jiang

**Affiliations:** 1 Research Institute of Climatic and Environmental Governance / Institute for Disaster Risk Management, School of Geographical Science, Nanjing University of Information Science & Technology, Nanjing 210044, China; 2 Pacific Global Solution, Nuku’alofa, Tongatapu, Tonga; 3 Global Energy Interconnection Development and Cooperation Organization, Beijing 100031, China; 4 Department of Environmental Engineering and Mechanical Engineering, Poznan University of Life Sciences, 60-637 Poznan, Poland; 5 Prime Minister Office of Western Samoa, Government of Samoa, Apia, Samoa; 6 Laboratory for Climate Risk and Urban-Rural Smart Governance / School of Geography, Jiangsu Second Normal University, Nanjing 210013, China

**Keywords:** environmental science, energy policy, engineering, energy engineering

## Abstract

Despite contributing less than 1% of global greenhouse gas (GHG) emissions, Small Island Developing States (SIDS) have the potential to drive global mitigation actions by advocating for ambitious emission reduction targets, promoting renewable energy solutions, and advancing sustainable development practices. The adoption of onshore-offshore wind and solar energy in 39 SIDS, which are currently experiencing the adverse effects of climate change, presents a significant opportunity. By harnessing renewable energy sources, these countries can effectively mitigate GHG emissions, enhance energy security, and build resilience. This approach aligns with the renewable energy roadmap outlined at the 28^th^ Conference of Parties (COP) of the United Nations Framework Convention on Climate Change (UNFCCC), facilitating a transition from fossil fuels to renewable energy sources. However, realizing such prospects requires collaboration among policymakers, industry stakeholders, and researchers to address multiple technical, economic, and environmental issues. Through this joint effort, the untapped potential of wind and solar energy can be fully harnessed, offering a pragmatic solution to actively mitigate climate change and the issues faced in these regions.

## Introduction

**Figure 1:**
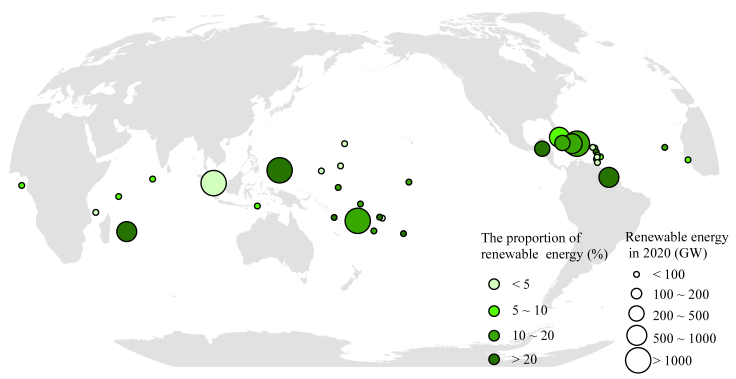
Location of the 39 SIDS examined in this paper and their aggregate characteristics concerning renewable energy Source of data: IRENA.^1^ Note: GW = gigawatts.

In the wake of escalating climate change impacts, 39 Small Island Developing States (SIDS) find themselves at the forefront of the global call to action for sustainable and resilient energy solutions. These 39 SIDS (Figure 1) are home to more than 68 million people distributed across a total land area of 1.16 million km^2^ and a total ocean coverage of Exclusive Economic Zones, EEZs, of approximately 12.65 million km^2^. They are uniquely positioned at the confluence of vulnerability and opportunity when it comes to global climate change. The primary economic engines of these 39 SIDS are often sectors that depend on climatic conditions and face immediate threats fronted by climate change.^1^^,^^2^^,^^3^^,^^4^^,^^5^^,^^6^^,^^7^

The SIDS studied in this paper follow the list given at the UN portal https://www.un.org/ohrlls/content/list-sids. It should be noted that, in fact, Guyana is not really an island and Guinea-Bissau is a coastal country on the African continent (including some small islands).

At the nexus of vulnerability and innovation lie these 39 SIDS, whose geographic and economic landscapes render them acutely repercussive to the harrowing effects of global climate change. These paradisiacal but endangered lands, characterized by their limited size, isolated geography, and susceptibility to natural disasters, are predominantly sustained by primary industries that are heavily dependent on the ecological balance—namely agriculture, tourism, and fisheries, which form the backbone of their gross domestic product.^1^^,^^3^^,^^4^^,^^5^

The SIDS are highly susceptible to the consequences of climate change and its impacts, which encompass escalating sea levels, coastal erosion, increased frequency and intensity of extreme weather events (EWEs), changes in precipitation patterns and water scarcity, increasing frequency and severity of occurrence of natural calamities, and risks to the variety of biodiversity loss, life, and ecosystem disruptions. If worldwide endeavors to diminish GHG emissions do not yield results promptly, SIDS might have to pivot their attention toward adjusting strategies to deal with these unavoidable transformations. It is of utmost importance for SIDS to prioritize fortifying their ability to withstand the repercussions of climate change by means such as enhancing infrastructure and early warning systems, and executing sustainable land use practices. By harmonizing both efforts to mitigate and adapt to climate change, SIDS can better equip themselves to confront the trials brought about by climate change and safeguard their societies and ecosystems for the forthcoming generations.

## Present status and prospects for the future

Table 1 lists all fossil carbon dioxide (CO_2_) emissions in the analyzed countries, in three categories – per capita, per country, and per energy unit. Fossil CO_2_ emissions per capita in SIDS countries range from 0.2 t (in Guinea-Bissau and Haiti) to 23.2 t in Trinidad and Tobago. A majority of examined countries have per capita emissions well below the global average of 4.7 t and only seven analyzed SIDS exceed this value. Emissions per unit of energy in individual SIDS range from 0.06 kg/kWh in Singapore to 0.38 kg/kWh in Papua New Guinea (PNG). Again, a majority of countries have per kWh emissions well below the global average of 0.22 kg/kWh, while 11 countries exceed this value. Emissions of CO_2_ per country range over three orders of magnitude, from 11 kt for Tuvalu (a country with a very small population that only slightly exceeds 11 thousand) to 53 Mt for Singapore.

**Table 1:** All fossil CO_2_ emissions in analyzed SIDS, per capita, per country, and per energy unit

Country name	Per capita (t/cap)	Per country (kt)	Per energy unit (kg/kWh)
**Asia-Pacific**			

Cook Islands	4.0	68	0.14
Fiji	1.2	1,000	0.13
Kiribati	0.5	68	0.22
Maldives	3.3	2,000	0.19
Marshall Islands	3.6	151	n.a.
Federated States of Micronesia	1.4	151	0.21
Nauru	4.3	53	0.20
Niue	3.9	7.5	0.24
Palau	12.3	220	n.a.
PNG	0.8	8,000	0.38
Samoa	1.2	249	0.16
Singapore	9.4	53,000	0.06
Solomon Islands∗	0.4	299	0.23
Tonga	1.8	189	0.27
Tuvalu∗	1.0	11	n.a.
Vanuatu	0.7	208	0.22

**Latin America and Caribbean**			

Antigua and Barbuda	6.4	602	0.22
Bahamas	5.1	2,000	0.15
Barbados	4.4	1,000	0.19
Belize	1.8	725	0.24
Cuba	1.9	21,000	0.20
Dominica	2.1	153	0.20
Dominican Republic	2.1	24,000	0.21
Grenada	2.7	340	0.28
Guyana	4.4	3,000	0.32
Haiti∗	0.2	2,000	0.21
Jamaica	2.3	6,000	0.19
Saint Kitts and Nevis	4.7	224	0.22
Saint Lucia	2.6	470	0.19
Saint Vincent and the Grenadines	2.3	239	0.27
Suriname	5.8	4,000	0.30
Trinidad and Tobago	23.2	34,000	0.21

**Africa**			

Cape Verde	1.0	569	0.13
Comoros∗	0.5	413	0.31
Guinea-Bissau∗	0.2	327	0.23
Mauritius	3.1	4,000	0.16
São Tomé and Príncipe∗	0.7	132	0.20
Seychelles	6.2	658	0.14
**Global mean**	4.7	–	0.22

Data represent 2021 or 2022, as available.

Source of data: https://ourworldindata.org/co2-and-greenhouse-gas-emissions.

Notation: n.a., number not available.

Tables 2 and 3 list installed wind and solar energy capacity, respectively, in SIDS in 2014–2023.

**Table 2:** Installed wind energy capacity in SIDS (in MW) in 2014–2023

Country	2014	2017	2020	2023
**Asia-Pacific**				

Fiji	10	10	10	10
Maldives	1	1	1	1
Samoa	1	1	1	1
Tonga	0	0	1	1

**Latin America and Caribbean**				

Cuba	16	16	16	16
Dominican Republic	80	135	370	417
Jamaica	42	102	102	102
St Kitts and Nevis	2	2	2	2

**Africa**				

Cabo Verde	27	27	27	27
Mauritius	1	11	11	11
Seychelles	6	6	6	6

Source: IRENA.^8^

For a number of SIDS (Singapore, Bahamas, Barbados, Belize, Dominicana, Grenada, Haiti, and Trinidad and Tobago), the installed wind energy capacity in 2014–2023 was equal to zero. For some SIDS, the numbers are not available in IRENA.^8^

Tables 2 and 3 show that in several SIDS there have been considerable increases in installed wind and energy capacity over the period 2014–2023. For instance, there was a massive, over 5-fold increase in installed wind energy capacity in the Dominican Republic, from a respectable 80 MW in 2014 to 417 MW in 2023 (Table 2). Even stronger in relative terms, an 11-fold increase was noted for Mauritius, from 1 to 11 MW. The increase in installed solar energy capacity was even more impressive (Table 3). For the Dominican Republic, the increase was over 71-fold, from 15 MW in 2014 to 1,077 MW in 2023 (higher absolute value of installed solar energy capacity than in any other SIDS). For Barbados, the increase was 69-fold: from 1 MW in 2014 to 69 MW in 2023. One should note the dynamic increase in both wind and solar energy capacity in Dominican Republic. Summing up both wind and solar energy, the capacity in the Dominican Republic increased from 95 MW in 2014 to 1,494 MW in 2023.

**Table 3:** Installed solar energy capacity in SIDS (in MW) in 2014–2023

Country	2014	2017	2020	2023
**Asia-Pacific**				

Cook Islands	2	3	6	6
Fiji	2	8	8	11
Kiribati	1	3	3	3
Maldives	4	11	27	37
Marshall Islands	1	2	2	2
Federated States of Micronesia	1	2	3	3
Nauru	0	1	2	3
Niue	0	0	1	1
Palau	1	2	3	20
PNG	0	1	3	4
Samoa	3	14	14	14
Singapore	26	116	332	901
Solomon Islands	1	3	2	4
Tonga	3	7	7	15
Tuvalu	0	4	4	4
Vanuatu	0	3	5	5

**Africa**				

Cabo Verde	6	6	15	26
Comoros	n.a.	1	1	4
Guinea-Bissau	0	1	2	2
Mauritius	3	33	108	108
São Tomé and Príncipe	0	0	0	0
Seychelles	1	4	8	17

**Latin America and Caribbean**				

Antigua and Barbuda	0	4	13	16
Bahamas	2	2	4	14
Barbados	1	16	49	69
Belize	0	1	1	7
Cuba	7	70	213	280
Dominica	0	1	0	0
Dominican Republic	15	95	386	1,077
Grenada	1	2	4	4
Haiti	2	3	3	4
Jamaica	4	56	107	110
St Kitts and Nevis	1	2	3	3
St Lucia	0	1	4	4
St Vincent and the Grenadines	1	3	4	5
Trinidad and Tobago	4	4	4	4

Source: IRENA.^8^

Table 4 lists the ten highest renewable energy targets by 2030 in SIDS’ national plans. If national 2030 targets in SIDS are met, they would sum up to 13 GW (13,000 MW).^1^ Many SIDS have committed to 100% renewable energy by 2030.^1^ National plans were established in different years (some of them, quite a long time ago). The plan of the Dominican Republic for 2030 has been largely surpassed already, many years earlier (cf. Tables 2 and 3).

**Table 4:** Ten highest renewable energy targets by 2030 in SIDS’ national plans

#	State	Planned energy target (in MW)
1	Cuba	2,144
2	PNG	2,116
3	Singapore	2,000
4	Jamaica	1,038
5	Mauritius	863
6	Dominican Republic	795
7	Barbados	625
8	Trinidad and Tobago	601
9	Antigua and Barbuda	483
10	Bahamas	471

Source: IRENA.^1^

Tables 2, 3, and 4 prove that examined SIDS have large potential for wind and solar energy, thus adding value to the prospects of global mitigation. The Dominican Republic and Singapore have already demonstrated effective strategies for achieving success. Table 4 shows that Papua New Guinea has considerable potential and ambitions (even if not yet materialized, cf. Tables 2 and 3). The country possesses extensive land and water resources compared to other SIDS in the Pacific region. This vastness presents the island with considerable potential to contribute to global climate change mitigation efforts, particularly through the development of onshore-offshore wind energy, as well as solar energy.

The shift toward utilizing both offshore and onshore wind, along with solar power, holds immense promise for the 39 SIDS in curbing climate change and transitioning their economies toward low-carbon outputs. These renewable resources enable them not only to diminish their dependence on fossil fuels but also to cut down on GHG emissions, thus aligning with worldwide initiatives to address climate change. Although SIDS account for less than 1% of the global GHG emissions,^8^^,^^9^ they harbor some of the world’s most climate-vulnerable populations.^1^^,^^3^^,^^4^^,^^5^^,^^10^^,^^11^^,^^12^^,^^13^^,^^14^^,^^15^

Moreover, embracing wind and solar power bolsters the energy independence of SIDS, given the plentiful and enduring nature of these resources. This shift not only mitigates the SIDS’ exposure to the volatile international fossil fuel market but also fortifies their defenses against the adverse impacts of climate-induced disasters.^1^^,^^3^^,^^4^^,^^5^^,^^6^^,^^7^

Additionally, the move toward wind and solar power is likely to catalyze economic growth within examined SIDS by fostering new sectors and generating employment opportunities in the realm of renewable energy. This could stimulate further investments, spur technological advancements, and promote local progress, thereby propelling sustainable development and reinforcing the economic stability of these countries.^1^^,^^3^^,^^4^^,^^5^^,^^6^

So, based on the aforementioned state of affairs, we strongly believe that the harnessing of both offshore and onshore wind energy, coupled with solar energy, offers a viable route for SIDS to fulfill their energy requirements in an eco-friendly and sustainable fashion. By tapping into largely untapped renewable sources, SIDS are poised to make a meaningful contribution to the global crusade against climate change and aid in the shift toward a greener, and more sustainable, future. This discourse aligns harmoniously with the goals set forth in significant international summits, including the 28^th^ Conference of Parties (COP) of the United Nations Framework Convention on Climate Change, aiming to foster robust strategies for climate change mitigation.^1^^,^^3^^,^^4^^,^^5^^,^^16^^,^^17^^,^^18^

The indispensable role of small island nations in confronting climate change is irrefutably etched into the contemporary environmental discourse. These microcosms of global issues showcase a profound capacity for demonstrating the effectiveness of renewable energy adoption, embodying sustainable development in action. As such, the deliberate and strategic harnessing of wind and solar energy in SIDS examined in the present perspective paper presents not only a pathway to safeguard their own economic and environmental future but also a beacon of light in the collective quest for global climate change mitigation.^5^^,^^19^^,^^20^^,^^21^^,^^22^

In the grand tapestry of international environmental efforts, the winds of change blow strongly from the shores of these 39 SIDS, carrying with them the promise of a more resilient and sustainable planet for all. By harnessing wind and solar energy, these countries could showcase their capacity for active mitigation, adding value to regional and global mitigation efforts and pathways.^1^^,^^2^^,^^4^

In the subsequent sections, we present a comprehensive explanation of how these countries could capitalize on wind and solar energy to showcase their capacity for active mitigation, thus adding value to the achievements of regional and global mitigation efforts and pathways.

## Onshore and offshore wind and solar energy in SIDS – Gaps and challenges

The 39 SIDS considered in this perspective article face unique challenges when it comes to transitioning to onshore and offshore wind and solar energy sources. This section explores the capacity gaps and challenges for these countries in their renewable energy transition.^1^^,^^3^^,^^7^^,^^23^^,^^24^^,^^25^^,^^26^

### Gaps and challenges

The transition to onshore and offshore wind and solar energy in 39 SIDS faces variable challenges due to limited land availability, geographic constraints, financial and technical limitations, intermittent and variable renewable energy sources, insufficient grid infrastructure, as well as limited local expertise and human capital.

#### Limited land availability

The SIDS typically have limited land area, making it challenging to find suitable locations for large-scale renewable energy installations. This restricts the capacity to harness wind and solar energy to its fullest potential.

#### Geographic constraints

The geographic location of SIDS, often isolated and far away from major onshore and offshore energy technology manufacturing hubs, presents logistical challenges and increased costs for importing necessary equipment, leading to capacity constraints.

#### Financial and technical constraints

**Figure 2:**
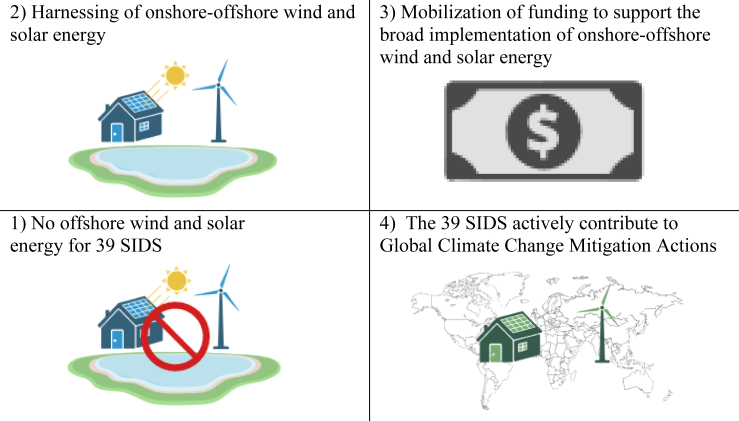
Onshore and offshore wind and solar energy challenges and opportunities for SIDS studied in this paper

The SIDS confront a multitude of difficulties when transitioning to renewable energy sources such as onshore and offshore wind and solar power. While certain countries prioritized onshore solar power exclusively, others failed to acknowledge onshore wind power altogether. Furthermore, the majority of SIDS exclusively considered onshore solar and wind power, overlooking the potential of offshore wind and solar power, which hindered their progress in renewable energy development. As a result, this exacerbates their situation and leads to an increase in challenges (Figure 2). These challenges can range from financial constraints to technical limitations, policy barriers, geographical locations, and access to international climate finance and the accounting for such finance.

#### Intermittency and variability of renewable energy sources

One of the significant challenges faced by SIDS in capitalizing on renewable energy is the intermittent and variable nature of wind and solar energy. The SIDS must overcome the technical and financial obstacles associated with energy storage systems to store excess renewable energy during periods of high production and use it during lower production periods.

#### Insufficient grid infrastructure

Many SIDS lack the adequate grid infrastructure necessary to accommodate the increased capacity and variability of renewable energy sources. Modernizing and expanding grid infrastructure is essential to fully capitalize on renewable energy potential and ensure a reliable and stable energy supply.

#### Limited local expertise and human capital

The SIDS often face a shortage of local expertise and human capital in the field of renewable energy. Developing and maintaining renewable energy projects require specialized skills, which may not be readily available in small island nations. Investing in education, training, and capacity-building programs can help address this challenge.

## Opportunities and tasks in renewable energy transition of SIDS and their role in reducing global climate change and its negative impacts

There are significant opportunities and tasks that SIDS could use to overcome the challenges discussed in the former section. Abundant renewable energy resources, climate change mitigation, regional collaborations, and technological innovations offer pathways for SIDS to enhance their renewable energy capacity and resilience.^1^^,^^3^^,^^4^^,^^5^^,^^7^^,^^24^^,^^25^^,^^26^

### Abundant renewable energy resources

Despite their small size, SIDS are blessed with abundant renewable energy resources such as solar, wind, and ocean currents, providing them with an opportunity for self-sufficiency and resilience. By harnessing these resources, SIDS can reduce dependence on imported fossil fuels and strengthen their energy security.

### Climate resilience and sustainable development

The SIDS are particularly vulnerable to the impacts of climate change, including rising sea levels and EWEs. Transitioning to renewable energy helps mitigate GHG emissions, reducing their contribution to climate change while promoting climate resilience and sustainable development.

### Regional collaboration and partnerships

Many SIDS share similar renewable energy challenges and opportunities. Regional collaboration and partnerships offer opportunities to pool resources, share experiences, and utilize economies of scale to overcome capacity challenges. Organizations like the Alliance of Small Island States and the International Renewable Energy Agency (IRENA) facilitate such collaborations.

### Technological innovations

Advancements in renewable energy technologies provide SIDS with access to innovative solutions. Compact solar panels, energy storage systems, and offshore wind turbines designed for limited land availability can bolster renewable energy capacity within SIDS. Collaborations with technology providers and research institutions can aid in customizing renewable energy solutions to suit the specific needs of SIDS (e.g., wind turbines with solar panels).

Continued support from international organizations, financial mechanisms, and technology transfer agreements would be crucial in realizing these opportunities and facilitating a sustainable energy future for SIDS.

Developments in renewable energy sectors in 39 SIDS considered here have been shaped and sustained by an intricate array of policies, strategic frameworks, and international support mechanisms, many of which are guided by the implementation of the Sustainable Development Goals (SDGs) 7 (renewable energy) and 13 (climate action). These advancements contribute to SDG 8, promoting economic growth and full employment, as the renewable sector powers job creation and fosters innovation through green tech industries.^17^^,^^27^

### Energy independence and security

Transitioning to renewable energy sources offers SIDS the opportunity to achieve energy independence and security. By reducing reliance on imported fossil fuels, SIDS can enhance energy resilience, reduce vulnerability to price fluctuations, and ensure a stable energy supply for their citizens. The IRENA has calculated that the 39 SIDS examined in this perspective paper could possess a total capacity of 10 GW of wind and solar energy by the year 2030. This capacity breakdown includes 2.3 GW for onshore wind, 2.3 GW for offshore wind, and 5.4 GW for solar energy. The exploitation of this untapped renewable energy potential can aid in global initiatives aimed at mitigating climate change.^1^^,^^4^^,^^17^^,^^18^ For the future, though, we found that these 39 SIDS have a lot of capacity in both wind and solar energy to offer for their own people.^17^^,^^18^^,^^23^^,^^27^

### Economic development and job creation

Investing in renewable energy presents an opportunity for economic development and job creation in SIDS. Renewable energy projects can attract direct foreign investment, create sustainable employment opportunities, and stimulate local economies. Additionally, the development and maintenance of renewable energy infrastructure can foster innovation and entrepreneurial activities.^17^^,^^18^^,^^23^^,^^27^

### Sustainable tourism and branding

Many SIDSs rely heavily on tourism as a significant source of revenue. By capitalizing on the renewable energy transition, SIDS can enhance its image as a sustainable tourism destination. The utilization of onshore and offshore wind farms and solar panels can significantly contribute to a green and eco-friendly brand, appealing to environmentally conscious tourists.^17^^,^^18^^,^^23^^,^^27^

### Climate change mitigation and adaptation

Renewable energy transition plays a crucial role in mitigating the negative impacts of global climate change on SIDS. By reducing GHG emissions associated with the use of fossil fuels, renewable energy would help these states meet their nationally determined contributions (NDCs) to global climate protection under the Paris Agreement. Moreover, the transition to renewable energy aligns with adaptation strategies, as it fosters resilience and reduces vulnerability to the impacts of climate change, such as sea-level rise and EWEs.^28^

## Actions for the transition to renewable energy with benefits to mitigation of and adaptation to climate change

The 39 SIDS studied in this perspective paper are actively working to create a structured approach for transitioning to renewable energy, intending to significantly reduce the detrimental consequences associated with climate change (Tables 2, 3, and 4).

This roadmap not only aligns with the contributions and targets of the 39 SIDS on their NDCs, but it also supports the objectives of the SIDS Decarbonization Forum^29^ as well as the SIDS Lighthouse Initiative project initiated by the IRENA.^1^^,^^3^^,^^4^ Furthermore, it takes into account the recommendations put forth by the Prime Ministers and the Governments of Samoa and Tonga. Based on this information, the outlined steps provide a general roadmap for the adoption of wind and solar energy in these 39 SIDS, serving as a comprehensive guide.^1^^,^^3^^,^^4^^,^^6^^,^^30^^,^^31^^,^^32^^,^^33^^,^^34^

It is important, however, to note that these steps may be subject to change, considering the specific context and needs of each SIDS. As a result, this plan is designed to be implemented by each SIDS in partnership with IRENA and any interested local, regional, or international agencies.

### Resource assessment

Comprehensive assessments of the wind and solar energy potential must be conducted by each SIDS, individually or collaboratively. This assessment can include mapping suitable placements for inland and seaward wind farms and solar installations, as well as utilizing advanced technologies and methodologies to analyze wind speed, solar irradiance, photovoltaic resources, and other relevant environmental factors.

### Policy and regulatory framework

Policy and regulatory frameworks must be developed and strengthened to support the integration of onshore-offshore wind and solar energy into the national energy mix. Doing so includes establishing clear targets for renewable energy deployment, introducing support incentives and feed-in tariffs, and streamlining the permitting and approval processes for renewable energy projects.

### Capacity building

Technical and institutional capacities must be enhanced to support the planning, development, and operation of wind and solar energy projects. Doing so involves providing training and knowledge-sharing opportunities for government officials, energy professionals, and stakeholders to build expertise in renewable energy technologies and project management.

### Stakeholder engagement

Engagement with local communities, industry stakeholders, and development partners must be established to ensure broad-based support and participation in the transition to renewable energy. Doing so includes conducting public consultations, fostering partnerships with the private sector, and leveraging international cooperation for knowledge exchange and financial support.

### Project development

The development of inland-seaward wind and solar energy projects must be facilitated by identifying suitable placements, designing sustainable installations, and procuring financing and investment. This work also involves conducting environmental impact assessments and ensuring the integration of renewable energy into the national energy development plans.

### Grid integration

The capacity and resilience of the existing energy grid must be enhanced to accommodate the increased deployment of wind and solar energy. Doing so includes upgrading and modernizing grid infrastructure, implementing smart grid technologies, and developing energy storage solutions to mitigate the intermittency and variability in renewable energy generation.

### Financing and investment

Domestic and international financial resources must be mobilized to support the deployment of onshore-offshore wind and solar energy. Doing so may involve leveraging climate finance, attracting private sector investment, and implementing innovative financing mechanisms such as green bonds and carbon credits.

### Community participation and empowerment

Local communities must be empowered to actively participate in and benefit from the development of wind and solar energy projects. This work includes creating opportunities for community ownership and involvement in renewable energy initiatives, promoting energy literacy and awareness, and ensuring inclusive and equitable access to the benefits of renewable energy, e.g., using the gender equality, disability and social inclusion framework.

### Robust monitoring and evaluation (M&E) framework

Robust M&E mechanisms must be established to track the performance and impact of shoreward-seaward wind and solar energy projects. Doing so involves collecting and analyzing relevant data on energy production, GHG emissions, socio-economic benefits, and other key indicators to inform evidence-based decision-making and continuous improvement.

### Lessons learned, knowledge sharing, and collaboration

Knowledge sharing, learning, and collaboration must be fostered at the national, regional, and international levels to exchange best practices, lessons learned, and technical expertise in the development and deployment of onshore-offshore wind and solar energy. Doing so includes participating in regional networks, engaging with international organizations, and contributing to the regional and global discourse on renewable energy and climate action, including presenting or reporting at every consecutive COP (that is 29^th^ in 2024, 30^th^ in 2025, and so on) and SDG evaluation in 2030.

## The projected effects of utilizing wind and solar energy in SIDS

With the threat of climate change looming large, the 39 SIDS analyzed in this perspective paper are intensifying their efforts to contribute to global climate change mitigation actions. By following through a series of actions stated earlier to overcome the challenges they face, these 39 SIDS can expect five main results that will not only benefit their own communities but also contribute significantly to global efforts to combat climate change.^24^^,^^34^^,^^35^^,^^36^

Firstly, by implementing renewable energy projects and transitioning toward a low-carbon economy, SIDS can significantly reduce their GHG emissions. This will lead to a decrease in their carbon footprint, which is crucial for meeting international climate targets such as the Paris Agreement. The reduction in emissions will help mitigate the impacts of climate change and contribute to global efforts to keep the earth’s temperature rise below 2°C (preferably 1.5°C)^32^^,^^37^^,^^38^ above the pre-industrial level.

Secondly, investing in renewable energy and energy efficiency measures will enhance the energy security of SIDS. Many SIDS are heavily dependent on imported fossil fuels for their energy needs, making them vulnerable to supply disruptions and price fluctuations. By shifting toward renewable sources of energy, such as solar, wind, and hydropower, SIDS can diversify their energy sources and reduce their reliance on imported fuels, thereby enhancing their energy security.^17^^,^^39^^,^^40^

Thirdly, embracing sustainable land use practices and promoting conservation efforts will help SIDS protect their natural ecosystems and biodiversity. Healthy ecosystems play a crucial role in sequestering carbon dioxide and regulating the climate. By safeguarding their forests, mangroves, and coral reefs, SIDS can enhance their resilience to climate change and protect the rich biodiversity that sustains their livelihoods.^41^^,^^42^

Fourthly, investing in climate-resilient infrastructure and disaster risk reduction measures will help SIDS adapt to the impacts of climate change, such as sea-level rise, EWEs, and ocean acidification. By building resilient infrastructure and implementing early warning systems, SIDS can reduce the risks posed by climate-related disasters and protect their communities from devastating impacts.^43^^,^^44^^,^^45^

Lastly, taking ambitious climate action can lead to socio-economic benefits for SIDS, such as job creation, improved public health, and enhanced food security. By prioritizing sustainable development and green growth, SIDS can create new green jobs in sectors like renewable energy, ecotourism, and sustainable agriculture. Additionally, reducing air pollution from fossil fuel combustion can lead to improved public health outcomes, while investing in climate-smart agriculture can enhance food security and reduce dependence on imported food.

Subsequently, through the enactment of a series of actions to address the challenges they encounter, the 39 SIDS can anticipate realizing significant results that will not only enhance the well-being of their own populations and societies but also contribute to global efforts in mitigating climate change. Embracing sustainable practices, investing in renewable energy sources, building resilience, and fostering green growth are fundamental steps that can assist 39 SIDS in securing a sustainable and prosperous future amid the impacts of climate change.^8^

Renewable energy transition plays a crucial role in mitigating the negative impacts of global climate change on SIDS. By reducing GHG emissions associated with the use of fossil fuels, renewable energy would help these states meet their NDCs under the Paris Agreement. Moreover, the transition to renewable energy aligns with adaptation strategies, as it fosters resilience and reduces vulnerability to the impacts of climate change, such as sea-level rise and EWEs.^28^

## Final remarks

This perspective paper highlights the substantial potential in the adoption of wind and solar energy by 39 SIDS, offering prospects of significant and multi-faceted benefits for society, economy, and the environment. Embracing renewable energy sources with appropriate policies and partnerships can sustainably meet the energy needs of these countries. Additionally, prioritizing GHG emission reduction and climate change adaptation aligns with global mitigation and adaptation goals. This transition also supports the COP 28 pledge, contributing to a low-carbon future, boosting energy security and economic sustainability, and improving access to energy resilience.^46^^,^^47^

As exemplars of a sustainable energy future, the 39 SIDS demonstrate the potential of onshore-offshore wind and solar energy in achieving both SDG 13 and the goals of COP 28,^17^^,^^18^^,^^27^ ensuring climate change mitigation and adaptation. Their experiences hold valuable lessons for the global community in advancing a resilient and clean energy future, contributing to the worldwide endeavor to expedite the shift from fossil fuels to renewable energy sources.

This perspective is based on the analysis and synthesis of perspectives contributed both by the existing literature and policy. We identify limitations that highlight the need for further research and analysis that take into account the unique characteristics and challenges of individual SIDS in order to effectively assess the potential contribution of onshore-offshore wind and solar energy to global climate change mitigation actions. The present study may not:

(1) Take into account the varying geographic, economic, and political characteristics of each SIDS. These characteristics can greatly influence the feasibility and effectiveness of onshore-offshore wind and solar energy implementation, as well as the overall impact on global climate change mitigation efforts.
(2) Adequately consider the specific energy needs and challenges faced by SIDS. These states often have limited resources, infrastructure, and technical expertise, which can impact the feasibility and scalability of renewable energy projects.
(3) Address the potential socio-economic implications of transitioning to onshore-offshore wind and solar energy. This includes potential impacts on traditional energy industries, job creation, energy access, and affordability for local communities.
(4) Consider the potential environmental impacts of large-scale onshore-offshore wind and solar energy projects, such as habitat destruction, land use changes, and wildlife displacement.

To surmount these limitations, it is proposed that in future research, the involvement of leaders from SIDS should be more actively sought. This proposal is underpinned by the existing literature and policy framework at present. Given the continuous evolution of these aspects, upcoming literature and policies are likely to address the knowledge deficiencies identified in this study. Nevertheless, researchers can adopt various measures based on the findings of this study.

(1) Collaborate with local experts: Working closely with local researchers, policymakers, and stakeholders can provide valuable insights into the specific challenges and opportunities of implementing renewable energy projects. Local expertise can help tailor the study to the unique context of each island state and ensure that recommendations are practical and culturally appropriate.
(2) Conduct comprehensive data collection: Gathering detailed information on energy consumption patterns, current energy infrastructure, economic conditions, and environmental considerations in SIDS is essential for accurate analysis. Using a combination of qualitative and quantitative data collection methods can provide a robust foundation for the study.
(3) Utilize modeling and simulation tools: Employing advanced modeling and simulation tools can help predict the potential impacts of wind and solar energy projects on GHG emissions, energy accessibility, and economic growth in SIDS. These tools can provide a more comprehensive understanding of the potential contributions of renewable energy to global climate change mitigation efforts.
(4) Consider a multi-disciplinary approach: Engaging experts from various disciplines such as engineering, economics, environmental science, and social sciences can offer diverse perspectives on the challenges and opportunities of renewable energy implementation in SIDS. A multi-disciplinary approach can lead to more holistic and well-rounded conclusions.
(5) Incorporate stakeholder engagement: Involving key stakeholders, including government officials, local communities, industry representatives, and non-profit organizations, throughout the research process can help ensure that the study addresses relevant concerns and incorporates local perspectives.

By implementing these strategies, researchers can enhance the credibility and applicability of the study on the potential contribution of wind and solar energy in SIDS to global climate change mitigation actions.

## Acknowledgments

The authors wish to acknowledge the financial support received from the International Cooperation Program between the 10.13039/501100001809National Science Foundation of China (NSFC) and the United Nations Environment Program (UNEP) under grant no. 42261144002. Additionally, they express gratitude for the support (scholarship for young researchers) provided to P.H.H. by the 10.13039/501100004543China Scholarship Council (CSC). Furthermore, the grant from the Global Energy Interconnection Development and Cooperation Organization is also recognized.

## Author contributions

Conceptualization, P.H.H., B.S., and T.J.; methodology, P.H.H., B.S., and T.J.; investigation, P.H.H., B.S., and T.J.; resources, P.H.H., B.S., and T.J.; writing – original draft, P.H.H. and B.S.; writing – review and editing, P.H.H., B.S., T.J., C.L., Z.W.K., Y.W., G.W., C.J., H.J., F.Y., and F.N.M.; supervision, B.S. and T.J.; project administration, B.S.; funding acquisition, B.S. and T.J.

## Declaration of interests

The authors declare no competing interests.
